# Extensional rheological data from ex-situ measurements for predicting porous media behaviour of the viscoelastic EOR polymers

**DOI:** 10.1016/j.dib.2018.07.066

**Published:** 2018-08-01

**Authors:** M.S. Azad, J.J. Trivedi

**Affiliations:** Department of Petroleum Engineering, University of Alberta, Edmonton, Canada

## Abstract

In this article, extensional rheological data of various polymer solutions, to be used in Azad Trivedi viscoelastic model (AT-VEM) for predicting the viscoelastic behavior of synthetic polymer in porous media are provided. Extensional rheology measurements are performed for different polymer solutions using Capillary breakup extensional Rheometer (CaBER) to obtain the filament diameter with respect to time. Extensional rheological parameters, such as the extensional relaxation time, maximum elongational viscosity at the critical Deborah number and strain hardening index are determined from observed filament diameter with time-based on the Upper Convected Maxwell model, the finite extensible non-linear elastic model, and the power law model.

**Specifications Table**

**Table:** 

Subject area	*Petroleum engineering, Reservoir engineering, Enhanced oil recovery, Chemical enhanced oil recovery*
More specific subject area	*Polymer flow through porous media, Viscoelastic Model, Extensional Rheology*
Type of data	*Figures and Table*
How data was acquired	*Capillary breakup extensional rheometer (CaBER) with an inbuilt laser micrometer*
Data format	*Measurement and Analyzed Data*
Experimental factors	*Polymer type, concentration, salinity of the brine*
Experimental features	*A small amount of polymeric solutions to be tested is loaded between the two plates of capillary breakup extensional rheometer. The top plate was separated from the bottom plate, thereby forming the filament. Filament drainage as a function of time was recorded by the inbuilt laser micrometer.*
Data source location	*EOR2S Lab, NREF 1–115, 9105 116 St NW, University of Alberta, Edmonton, Canada,*
Data accessibility	*The data is available within the article*
Related research article	This data in brief article is submitted as a companion paper to Azad and Trivedi [1].

**Value of the data**

• These data are valuable for the flow of polymer through porous media
• The data presented here shows, for the first time, the direct measurement of extensional rheological parameters of viscoelastic enhanced oil recovery (EOR) polymers.
• The data presented here shows the effect of salinity, concentration and molecular weight on the extensional rheological parameters of high molecular weight synthetic polymers, mainly hydrolyzed polyacrylamide.
• The data of extensional relaxation time, maximum elongational viscosity at the critical Deborah number and strain hardening index obtained from filament drainage as a function of time can be used to screen polymers for EOR as well as fracturing applications.
• The presented data validates the Azad-Trivedi Viscoelastic Model (AT-VEM) and compares with Unified Viscosity Model (UVM) for predicting the viscoelastic onset and shear thickening for polymer flow through porous media using only bulk rheology.
• The data presented can be used to predict the injectivity behavior and oil recovery potential of viscoelastic polymers using AT-VEM, independent of core flood experimental parameters.

## 1 Data

The extensional rheological data of different EOR polymers, measured using CaBER at various conditions of concentration and salinity are provided in this article. Initially, the reduction in filament diameter as a function of time was monitored using the inbuilt laser micrometer. Extensional parameters such as extensional relaxation time ($\tau_{ext}$), maximum elongational viscosity at critical Deborah number ($\mu_{\max\;@De_{cr-0.66}}$) and strain hardening index (*n*_*2*_) are determined from filament diameter with respect to time data using upper convected Maxwell (UCM) model, finite extensible non-linear elastic (FENE) model, and the power law model. These parameters are used in the AT-VEM for predicting the viscoelastic characteristics (such as onset, shear thickening) of EOR polymers [1]. The predictability of core flood independent AT-VEM is compared with UVM and Carreau model [1]. The details about UVM and Carreau model can be found elsewhere [2], [3].

## 2 Experimental design, materials and methods

### 2.1 Polymer preparation

HPAM 3230, HPAM 3530, HPAM 3630 and pusher 700 polymers were obtained from SNF Floerger (USA). Hengfloc 63020 and Hengfloc 63026 were obtained from Henju Beijing (China). The properties of the polymer, used in the various experiments are provided in Table 1. All the polymer solutions were prepared by stirring at 200 rpm for 24 h.

**Table 1:** Measured extensional parameters of various polymers.

Data set	Polymer	Concentration (ppm)	Molecular weight (million Daltons)	Salinity	$\tau_{ext}$(s)	$\mu_{\max\;@De_{cr-0.66}}$(cP)	*n*_*2*_	*n*
1	HPAM 3630	1500	18–20	20,040 ppm (640 ppm Ca2+ ions)	0.086	147,000	3.520	0.755
2	HENGFLOC 63020	1500	20	20,040 ppm (640 ppm Ca2+ ions)	0.048	26,000	3.484	0.828
3	HENGFLOC 63020	1500	20	20,040 ppm (20 ppm Ca2+ ions)	0.107	165,000	3.586	0.72
4	HENGFLOC 63026	1500	26	20,040 ppm (20 Ca2+ ions)	0.146	285,000	3.597	0.662
5	Pusher 700	1000	8	10,000 ppm	0.032	37,000	3.372	0.75
6	Pusher 700	1000	8	1000 ppm	0.0623	48,000	3.029	0.6
7	Pusher 700	1000	8	10,000 ppm	0.032	37,000	3.372	0.72
8	HPAM 3630	850	18–20	20,000 ppm	0.198	250,000	3.975	0.49
9	HPAM 3630	850	18–20	10,000 ppm	0.216	340,000	4.092	0.42
10	HPAM 3630	600	18–20	10,000 ppm	0.169	220,000	3.948	0.5
11	HPAM 3230	2500	6–8	25,200 ppm	0.0371	37,000	3.602	0.7
12	HPAM 3530	200	16–17	0 ppm	0.073	30,000	2.989	0.58
13	HPAM 3630	500	18–20	21,963 ppm	0.097	160,000	3.531	0.483
14	HPAM 3630	500	18–20	21,963 ppm	0.097	160,000	3.531	0.483

### 2.2 CaBER experimental procedure

HAAKE CaBER (Thermo Scientific, USA) was used for characterizing the extensional properties of EOR polymers. The details about the CaBER experimentation can be found elsewhere [1], [4], [5], [6]. The typical filament drainage schematic [4] during CaBER experimentation is shown in Fig. 1. The filament drainage (filament diameter decreases with time) for the polymer solutions used are shown in the Figs. 2(a)–15(a). Theories used to determine the extensional rheological data are briefed here, however, more details can be found in our previous publications [1], [4], [5], [6].

**Fig. 1:**
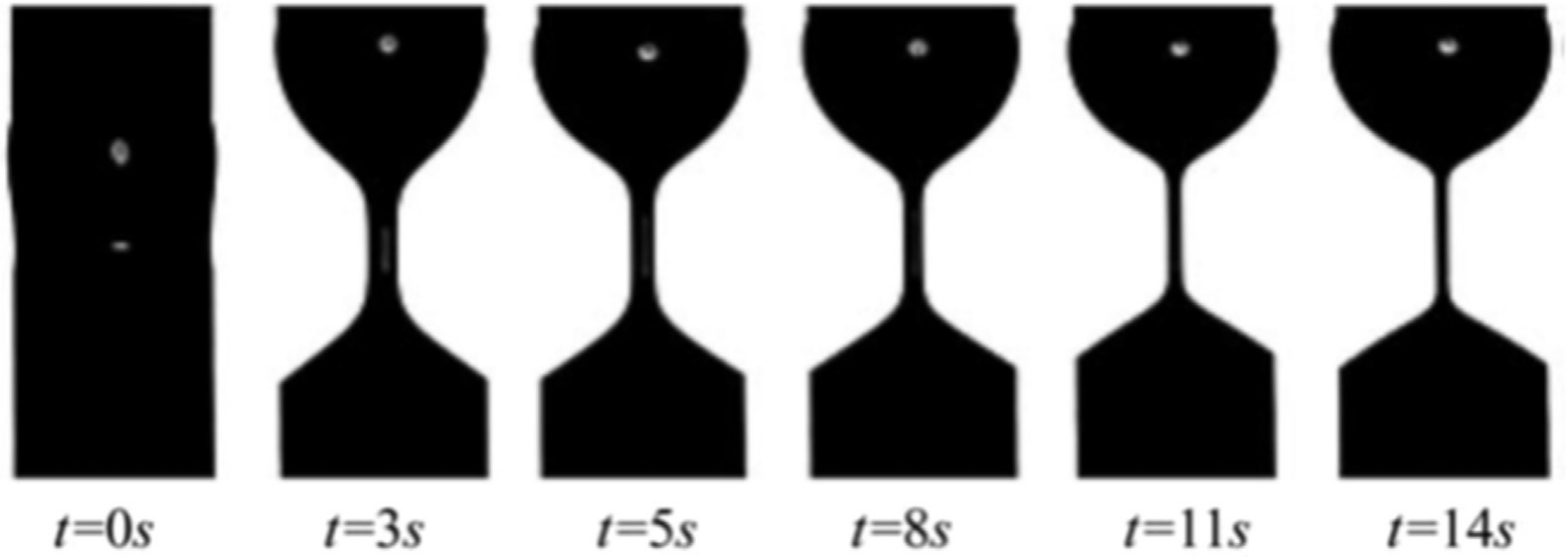
–Schematic describing the filament drainage (Azad et al. [4]).

**Fig. 2:**
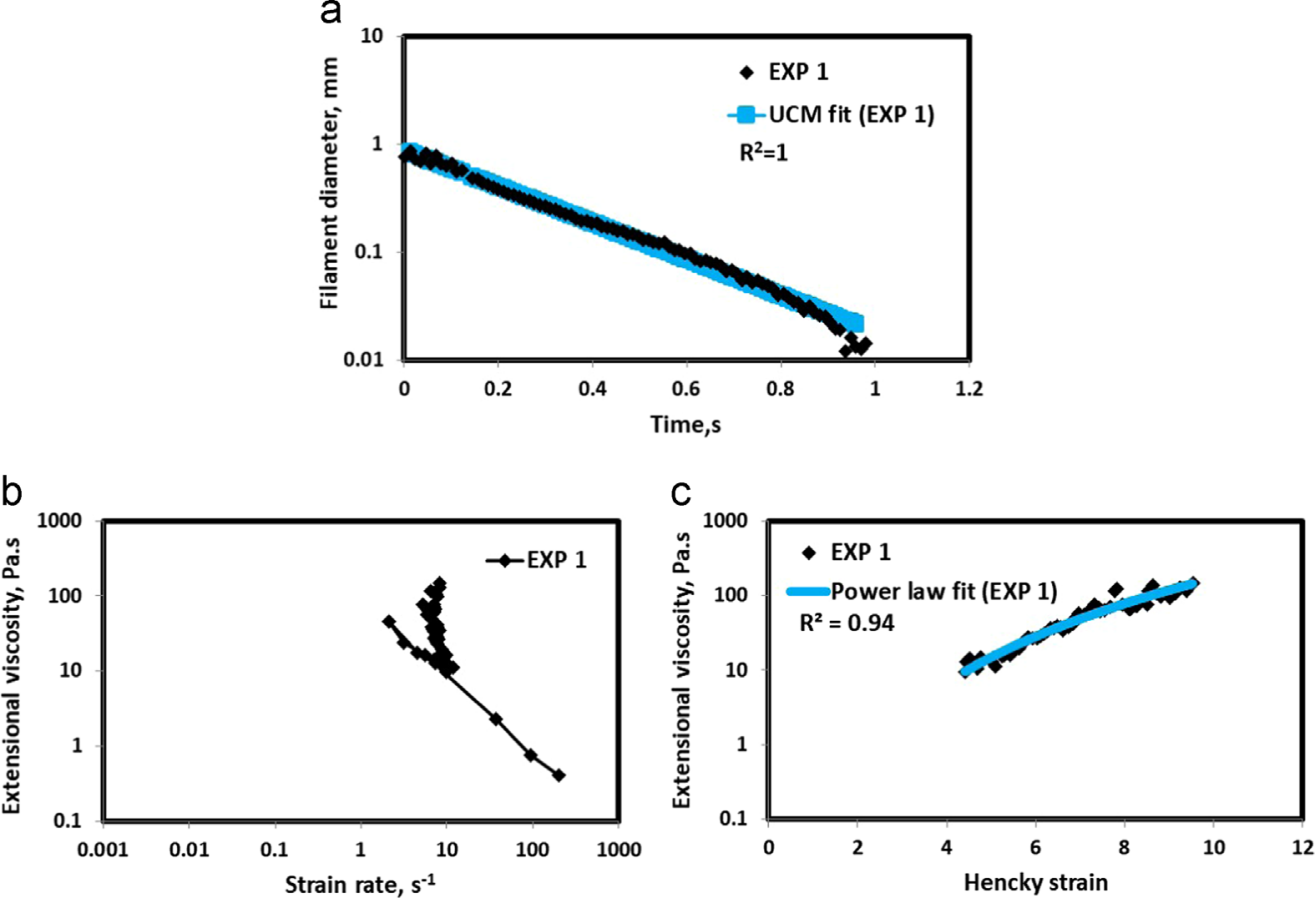
(a) Filament diameter vs time plot for EXP 1 and the UCM fit to the linear elastic regimes for the determination of relaxation time (b) Extensional viscosity as a function of generated strain rate plot showing the sharp rise in the extensional viscosity around the critical Deborah number (c) Power law fit to the extensional viscosity vs Hencky strain values around the critical Deborah number for the determination of strain hardening index.

**Fig. 3:**
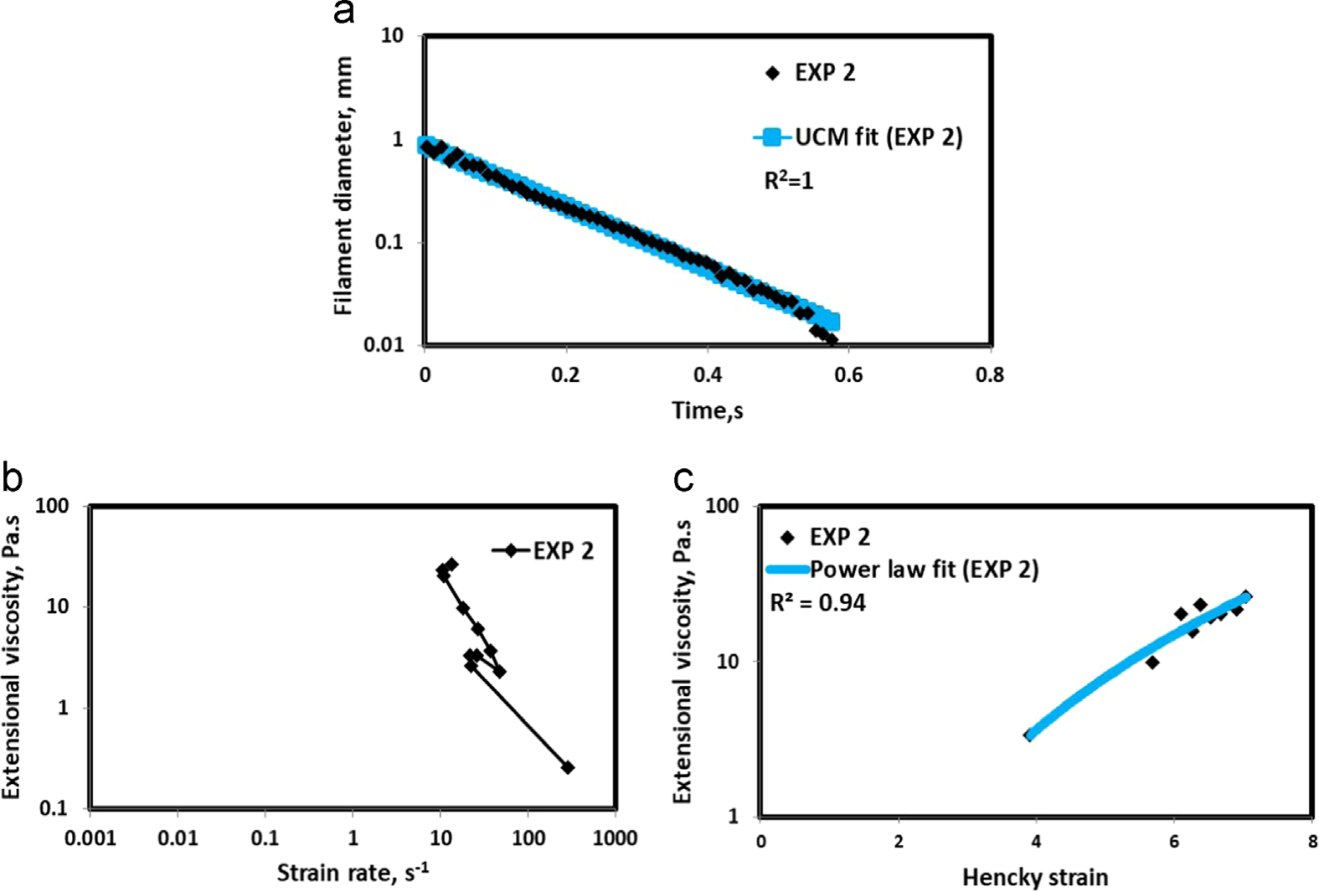
(a) Filament diameter vs time plot for EXP 2 and the UCM fit to the linear elastic regimes for the determination of relaxation time (b) Extensional viscosity as a function of generated strain rate plot showing the sharp rise in the extensional viscosity around the critical Deborah number (c) Power law fit to the extensional viscosity vs Hencky strain values around the critical Deborah number for the determination of strain hardening index.

**Fig. 4:**
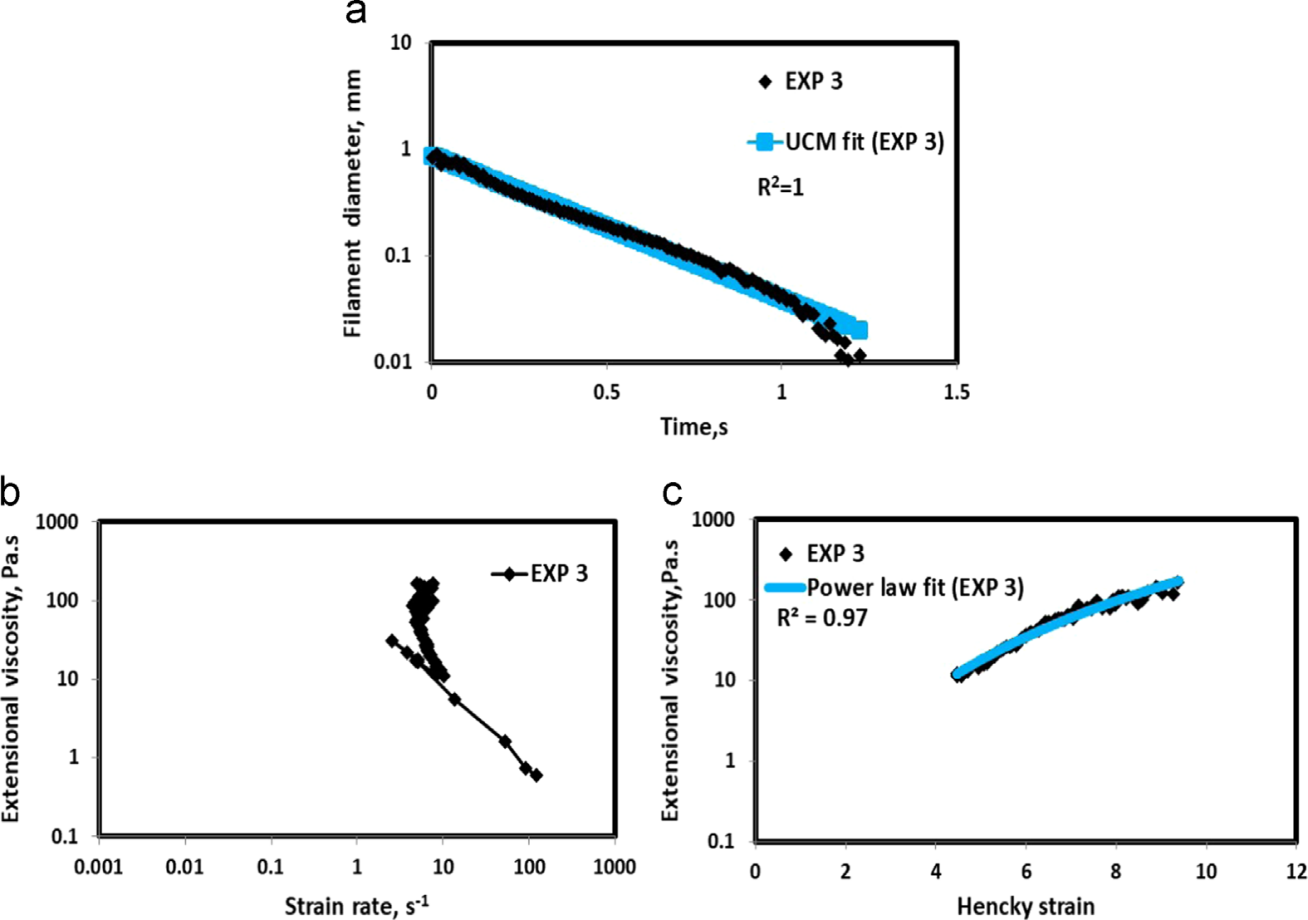
(a) Filament diameter vs time plot for EXP 3 and the UCM fit to the linear elastic regimes for the determination of relaxation time (b) Extensional viscosity as a function of generated strain rate plot showing the sharp rise in the extensional viscosity around the critical Deborah number (c) Power law fit to the extensional viscosity vs Hencky strain values around the critical Deborah number for the determination of strain hardening index.

**Fig. 5:**
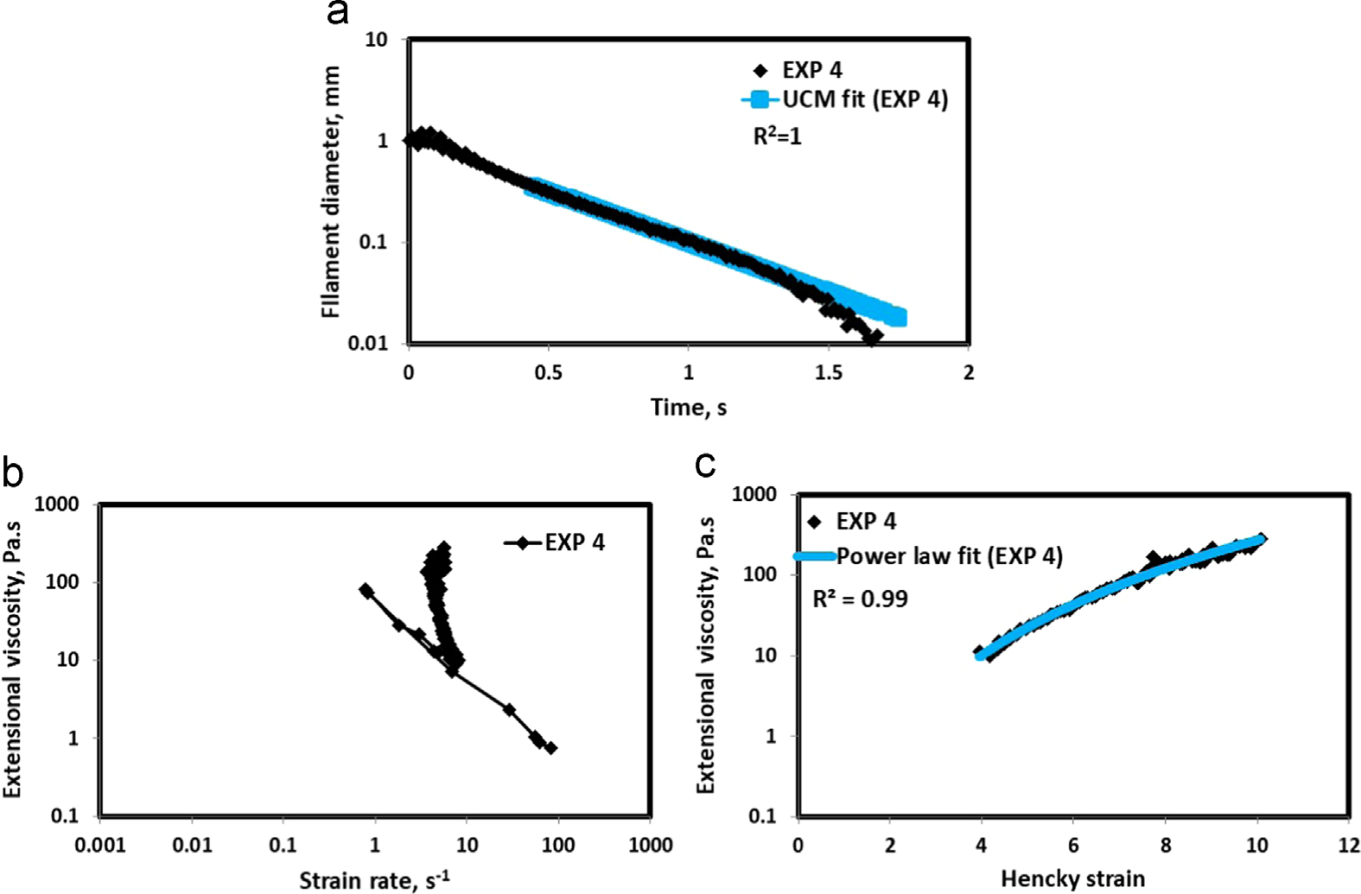
(a) Filament diameter vs time plot for EXP 4 and the UCM fit to the linear elastic regimes for the determination of relaxation time (b) Extensional viscosity as a function of generated strain rate plot showing the sharp rise in the extensional viscosity around the critical Deborah number (c) Power law fit to the extensional viscosity vs Hencky strain values around the critical Deborah number for the determination of strain hardening index.

**Fig. 6:**
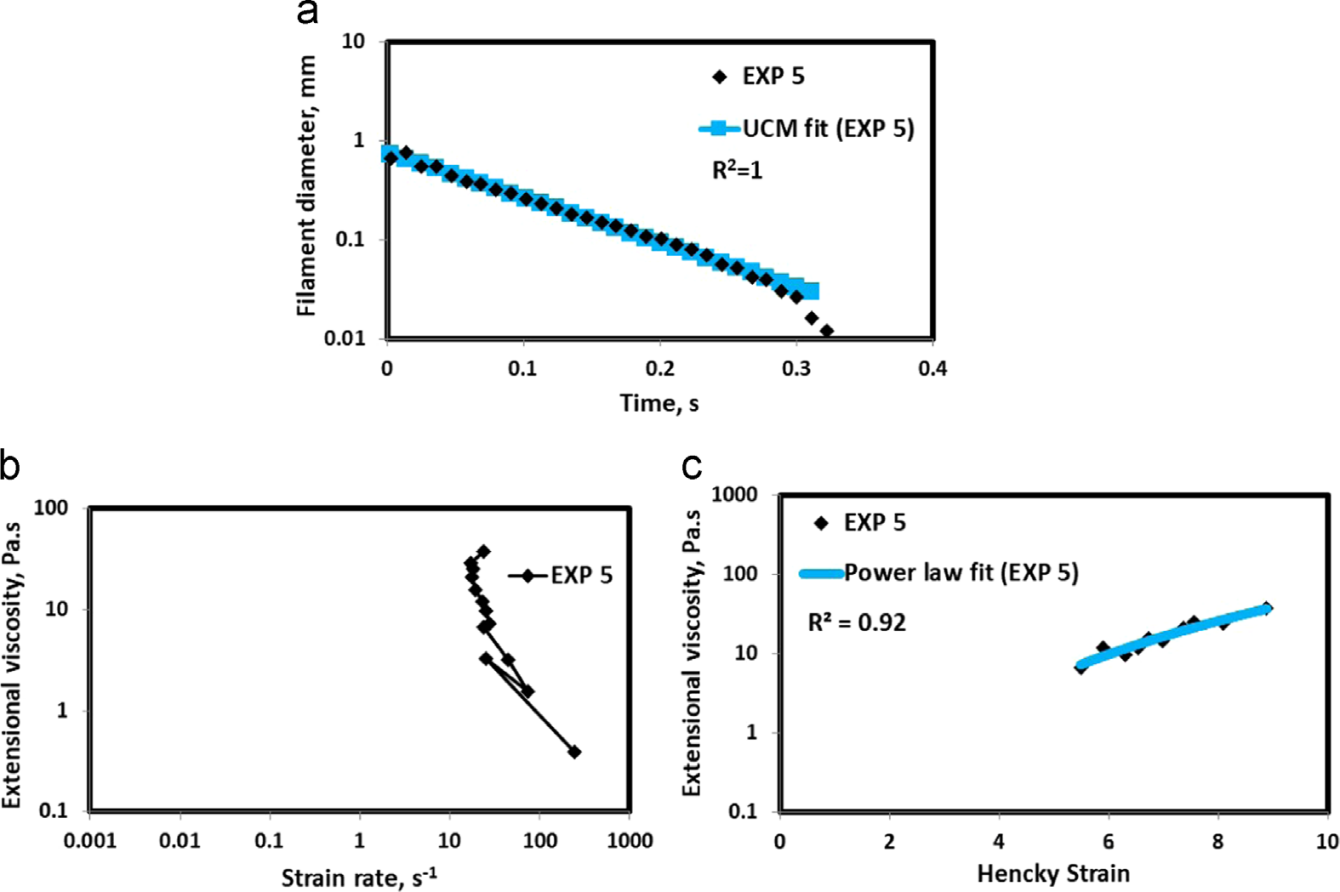
(a) Filament diameter vs time plot for EXP 5 and the UCM fit to the linear elastic regimes for the determination of relaxation time (b) Extensional viscosity as a function of generated strain rate plot showing the sharp rise in the extensional viscosity around the critical Deborah number (c) Power law fit to the extensional viscosity vs Hencky strain values around the critical Deborah number for the determination of strain hardening index.

**Fig. 7:**
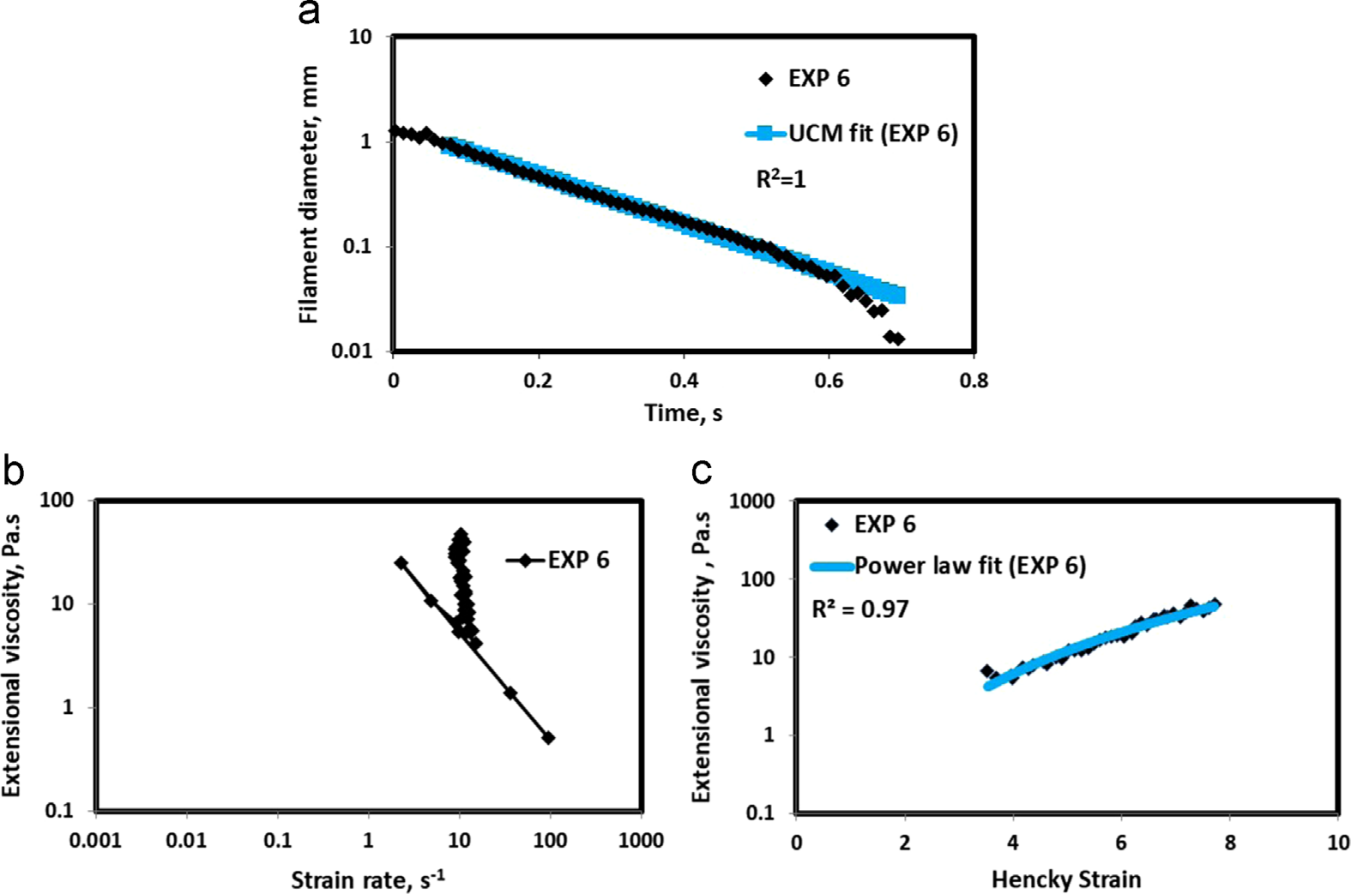
(a) Filament diameter vs time plot for EXP 6 and the UCM fit to the linear elastic regimes for the determination of relaxation time (b) Extensional viscosity as a function of generated strain rate plot showing the sharp rise in the extensional viscosity around the critical Deborah number (c) Power law fit to the extensional viscosity vs Hencky strain values around the critical Deborah number for the determination of strain hardening index.

**Fig. 8:**
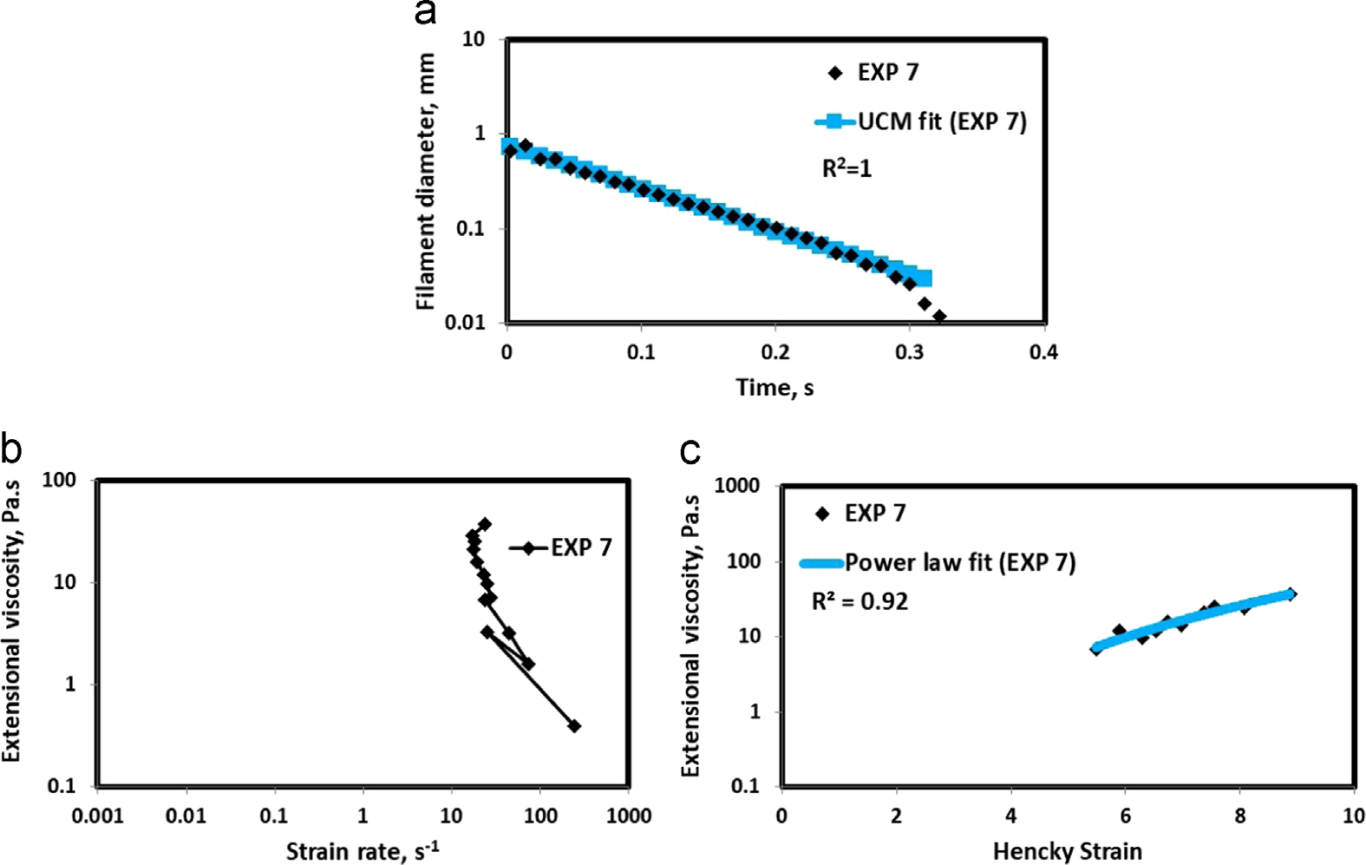
(a) Filament diameter vs time plot for EXP 7 and the UCM fit to the linear elastic regimes for the determination of relaxation time (b) Extensional viscosity as a function of generated strain rate plot showing the sharp rise in the extensional viscosity around the critical Deborah number (c) Power law fit to the extensional viscosity vs Hencky strain values around the critical Deborah number for the determination of strain hardening index.

**Fig. 9:**
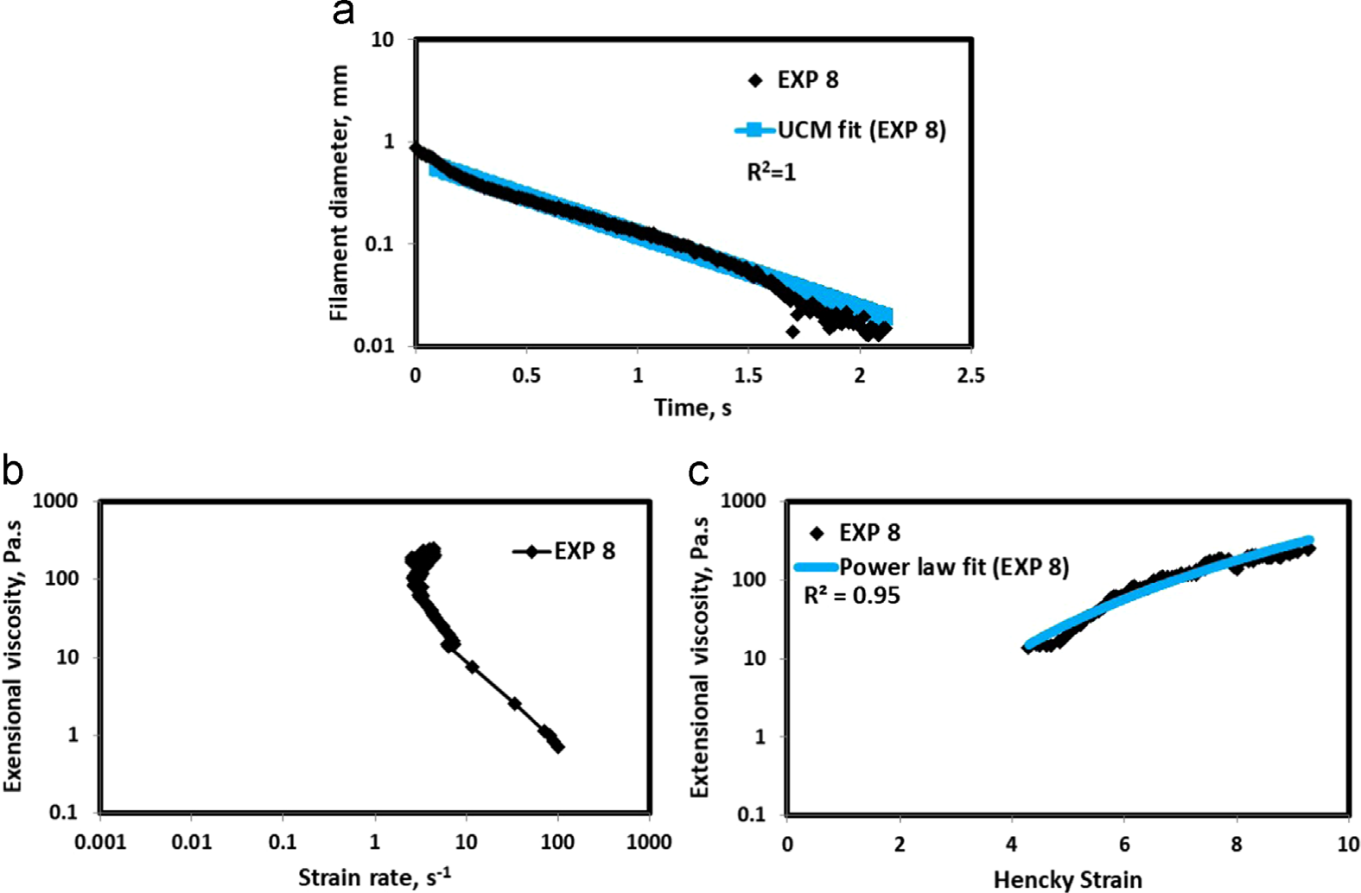
(a) Filament diameter vs time plot for EXP 8 and the UCM fit to the linear elastic regimes for the determination of relaxation time (b) Extensional viscosity as a function of generated strain rate plot showing the sharp rise in the extensional viscosity around the critical Deborah number (c) Power law fit to the extensional viscosity vs Hencky strain values around the critical Deborah number for the determination of strain hardening index.

**Fig. 10:**
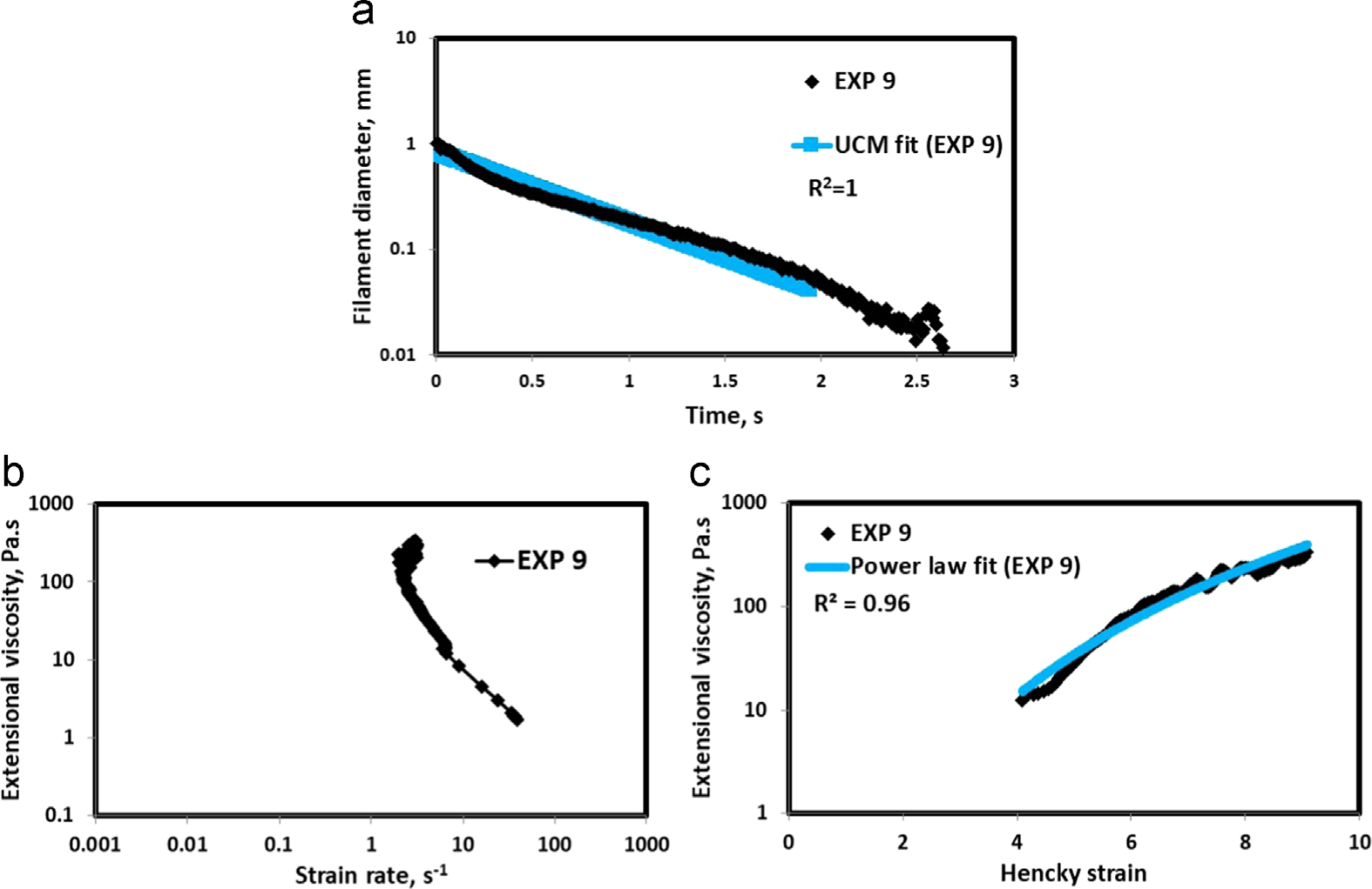
(a) Filament diameter vs time plot for EXP 9 and the UCM fit to the linear elastic regimes for the determination of relaxation time (b) Extensional viscosity as a function of generated strain rate plot showing the sharp rise in the extensional viscosity around the critical Deborah number (c) Power law fit to the extensional viscosity vs Hencky strain values around the critical Deborah number for the determination of strain hardening index.

**Fig. 11:**
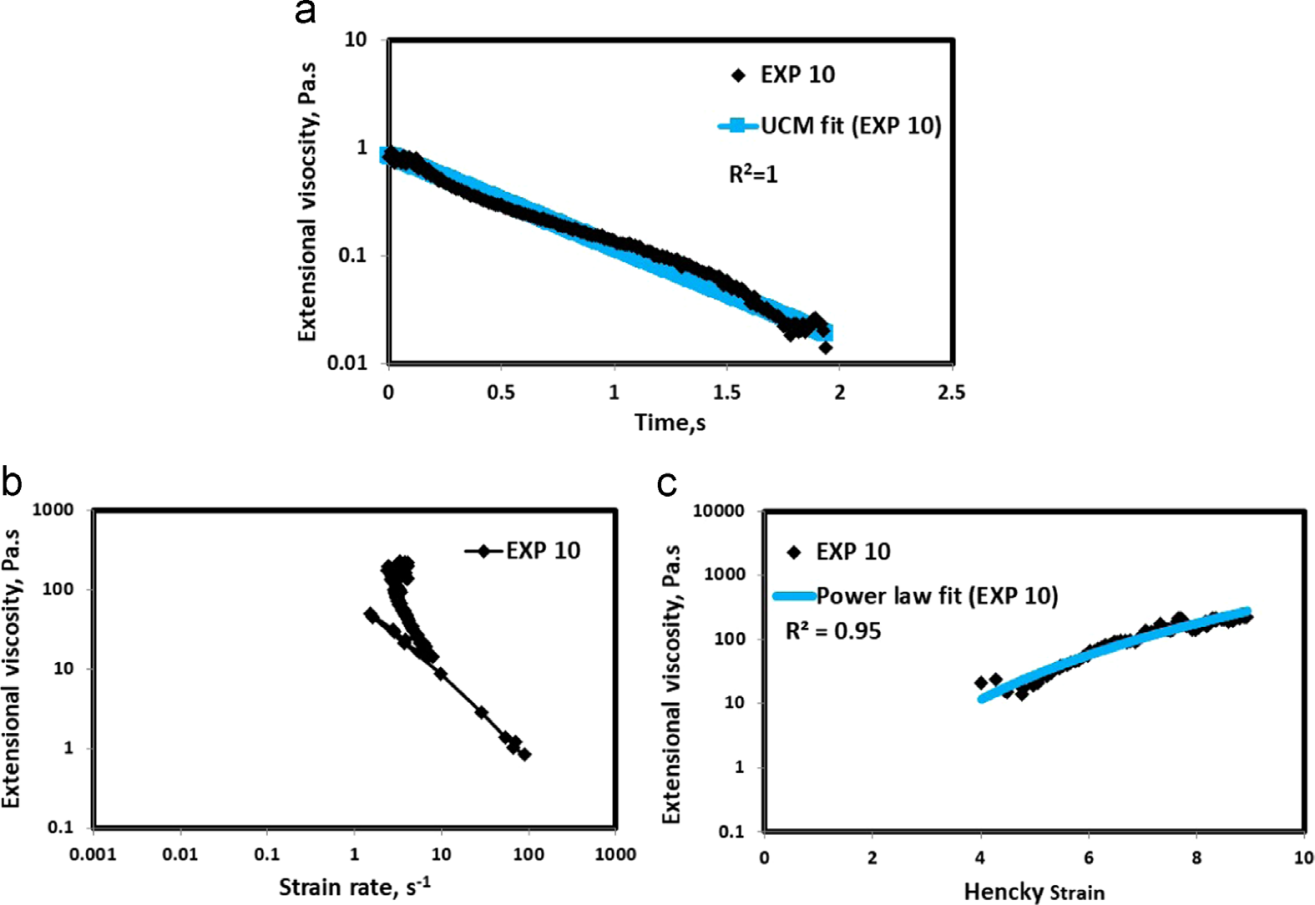
(a) Filament diameter vs time plot for EXP 10 and the UCM fit to the linear elastic regimes for the determination of relaxation time (b) Extensional viscosity as a function of generated strain rate plot showing the sharp rise in the extensional viscosity around the critical Deborah number (c) Power law fit to the extensional viscosity vs Hencky strain values around the critical Deborah number for the determination of strain hardening index.

**Fig. 12:**
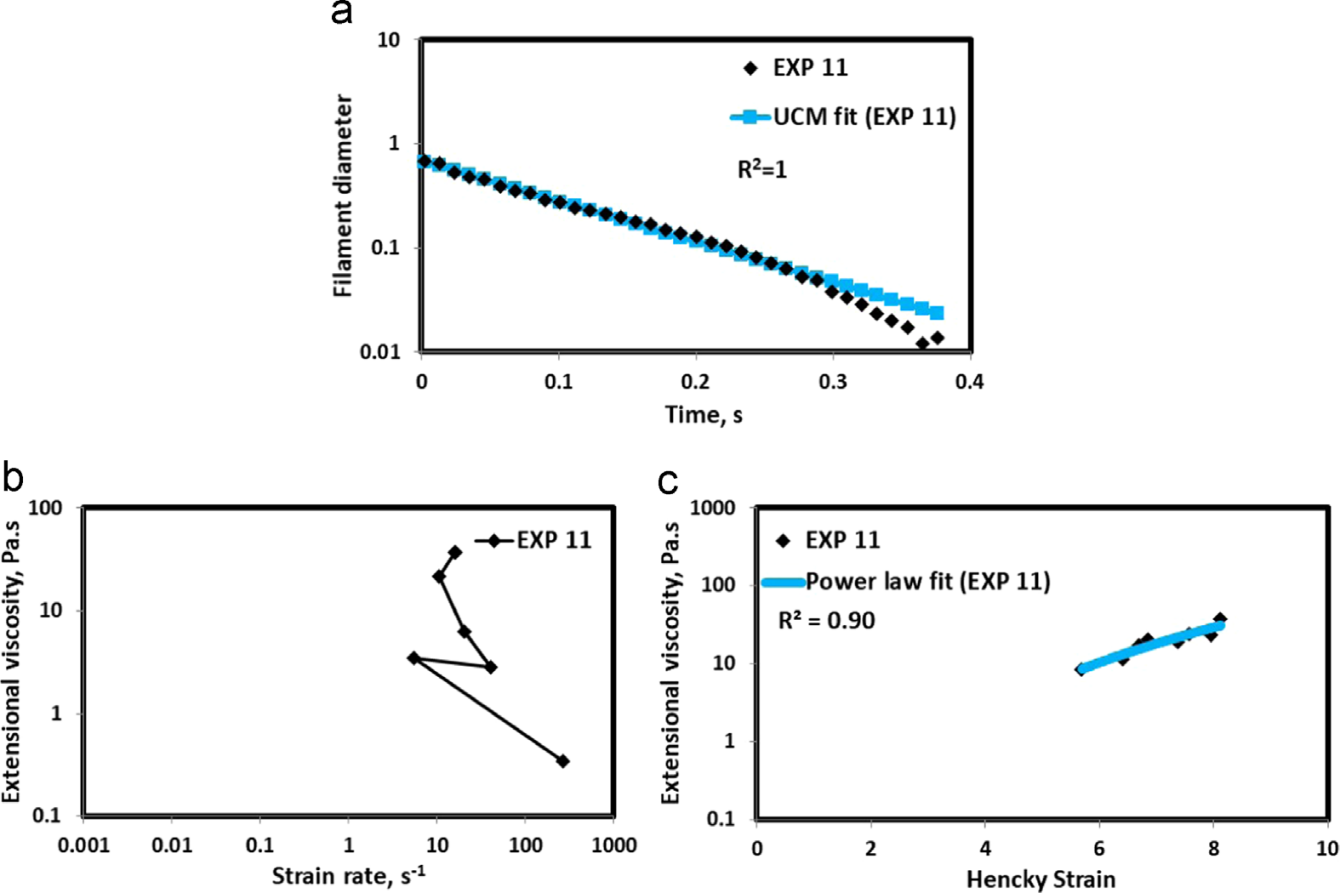
(a) Filament diameter vs time plot for EXP 11 and the UCM fit to the linear elastic regimes for the determination of relaxation time (b) Extensional viscosity as a function of generated strain rate plot showing the sharp rise in the extensional viscosity around the critical Deborah number (c) Power law fit to the extensional viscosity vs Hencky strain values around the critical Deborah number for the determination of strain hardening index.

**Fig. 13:**
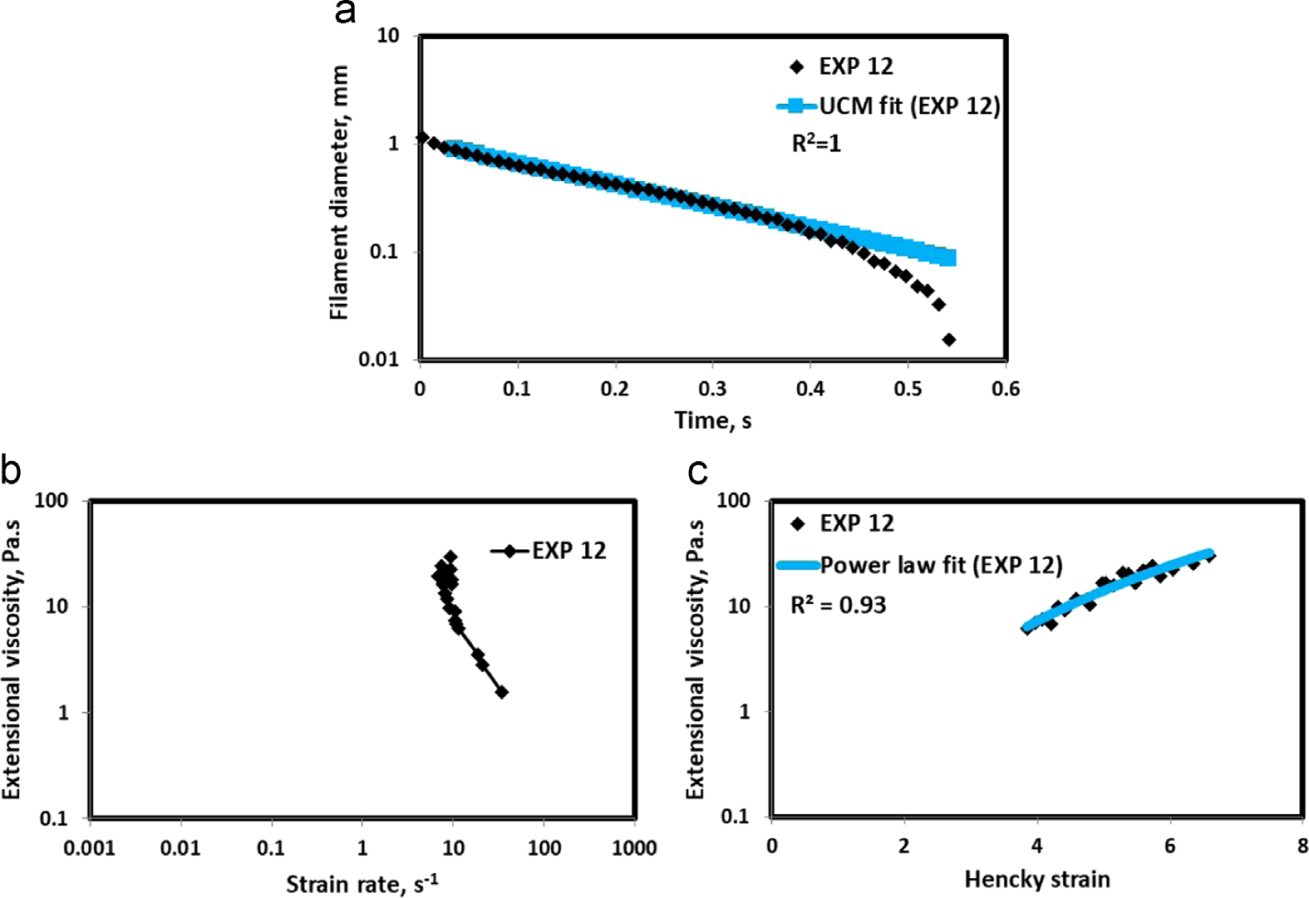
(a) Filament diameter vs time plot for EXP 12 and the UCM fit to the linear elastic regimes for the determination of relaxation time (b) Extensional viscosity as a function of generated strain rate plot showing the sharp rise in the extensional viscosity around the critical Deborah number (c) Power law fit to the extensional viscosity vs Hencky strain values around the critical Deborah number for the determination of strain hardening index.

**Fig. 14:**
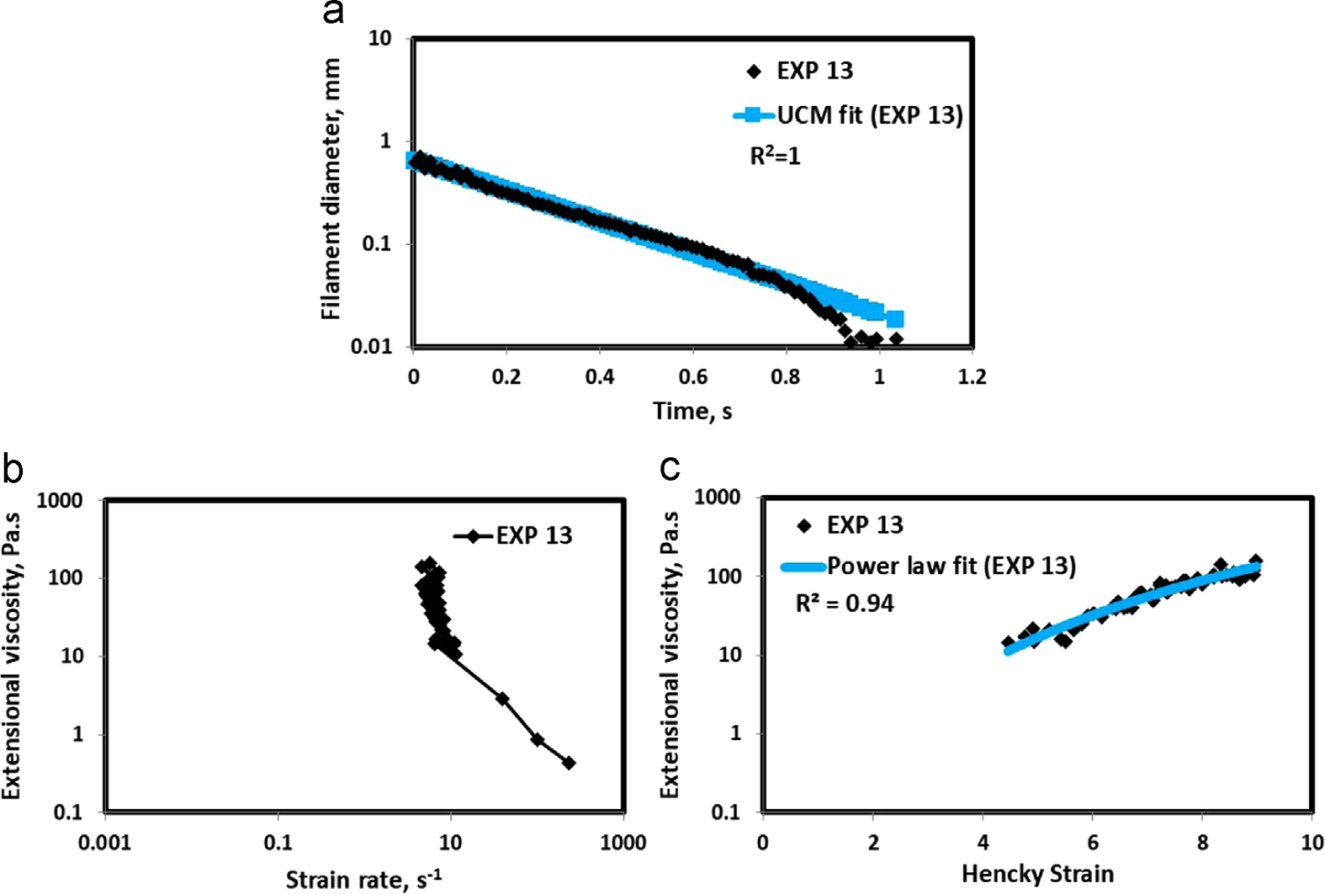
(a) Filament diameter vs time plot for EXP 13 and the UCM fit to the linear elastic regimes for the determination of relaxation time (b) Extensional viscosity as a function of generated strain rate plot showing the sharp rise in the extensional viscosity around the critical Deborah number (c) Power law fit to the extensional viscosity vs Hencky strain values around the critical Deborah number for the determination of strain hardening index.

**Fig. 15:**
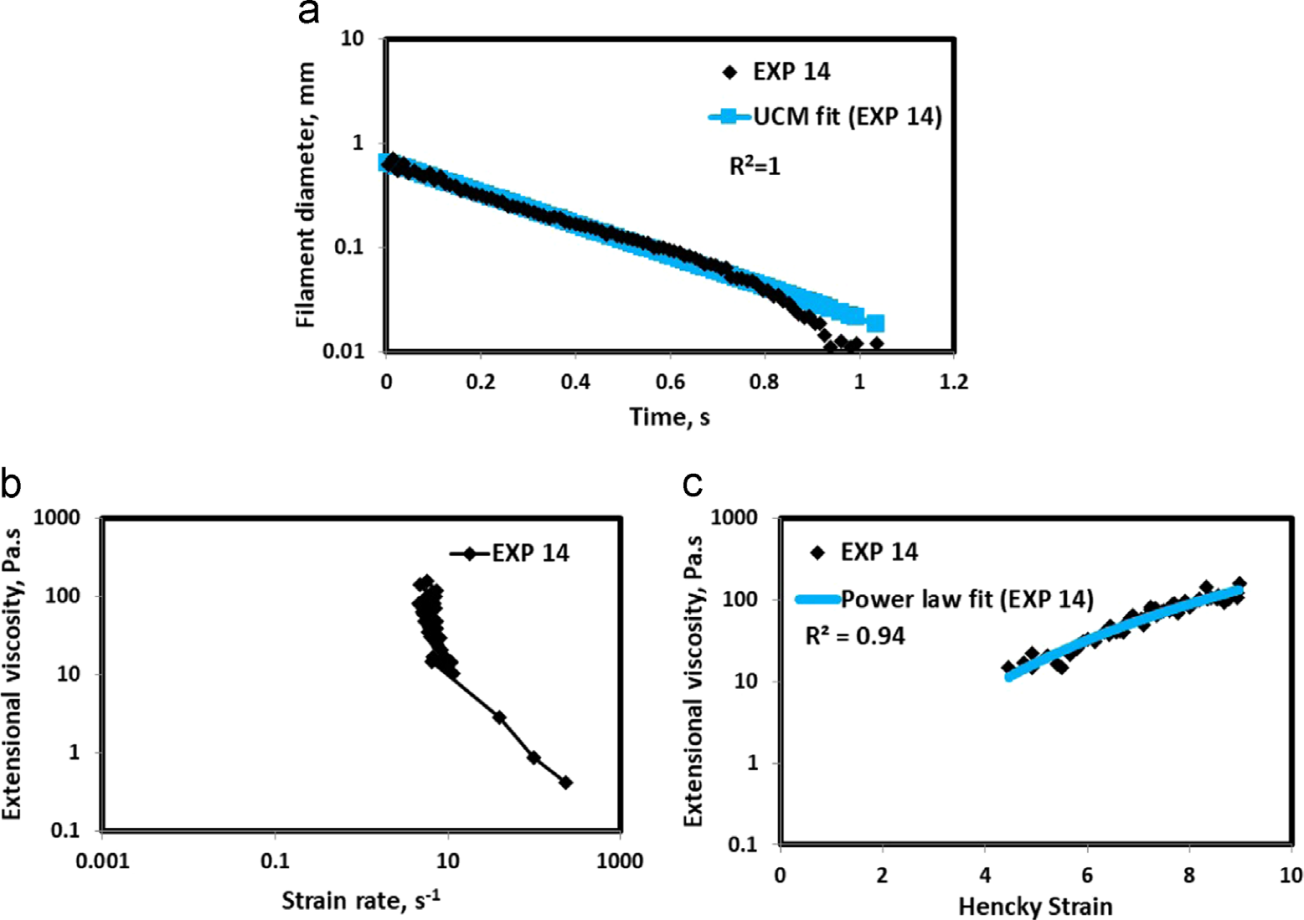
(a) Filament diameter vs time plot for EXP 14 and the UCM fit to the linear elastic regimes for the determination of relaxation time (b) Extensional viscosity as a function of generated strain rate plot showing the sharp rise in the extensional viscosity around the critical Deborah number (c) Power law fit to the extensional viscosity vs Hencky strain values around the critical Deborah number for the determination of strain hardening index.

#### 2.2.1 UCM model for extensional relaxation time

UCM model (Eq. (1)) is fitted to the linear part of the filament diameter vs time data in a semi-log plot to determine$\tau_{ext}$. The extracted and fitted data of all the polymer solutions, are highlighted by the blue line (Figs. 2(a)–15(a)). The average value of $\tau_{ext}$ is calculated from slope using the Eq. (1). The surface tension of water (73 milli N/m) is used for all the solutions. The calculated values of $\tau_{ext}$ are shown in Table 1.

(1) $$D_{mid}\left(t\right)=D_{o}{\left(\frac{G*D_{o}}{4*\sigma }\right)}^{\frac{1}{3}}e^{\left(\frac{-t}{3*\tau_{ext}}\right)}$$

where,

$D_{mid}(t)$ = mid-point diameter, mm
$D_{o}$ = initial diameter of sample, mm
G = Elastic modulus, Pa
$\tau_{ext}$= Extensional relaxation time, s

#### 2.2.2 FENE theory for maximum elongational viscosity

The axial force balance, strain rate and strain pertinent to filament drainage in CaBER experiments are represented by the Eqs. (2), (3), (4).

(2) $$\frac{2\sigma }{D_{mid}}=3\eta_{\varepsilon }\dot{\varepsilon }+\left(\tau_{zz}-\tau_{rr}\right)$$

where,

$\eta_{\varepsilon }$ = Solvent viscosity, Pa s

$\tau_{\mathrm{zz}}$ = First Normal stress, Pa

$\tau_{\mathrm{rr}}$ = Second normal stress, Pa

$\dot{\varepsilon }$ = Strain rate s^−1^

The strain and strain rate is calculated using the Eqs. (3), (4)

(3) $$\varepsilon \left(t\right)=2\;\ln\left(\frac{D_{o}}{D_{mid\left(t\right)}}\right)$$

(4) $$\dot{\varepsilon }\left(t\right)=\frac{-2}{D_{mid(t)}}\left(\frac{dD_{mid(t)}}{dt}\right)$$

where,

ε = Hencky strain, dimensionless
$\dot{\varepsilon }$ = Strain/Elongation rate, s^−1^

The elongational viscosity derived by substituting Eq. (4) into Eq. (2) is represented by Eq. (5).

(5) $$\eta_{app}\left(e\right)=-\frac{\left(2x-1\right)\sigma }{\frac{dD_{mid}}{dt}}$$

where,

η= Newtonian viscosity, Pa s
$\tau_{zz}-\tau_{rr}$ = Normal stress difference
ε(t) = Strain
$\dot{\varepsilon }\left(t\right)$ = Strain rate, s^−1^
$D_{mid}\left(t\right)$ = Mid-point diameter, mm
$\eta_{app}\left(e\right)$ = Apparent extensional viscosity, Pa s
X = Correction factor for axial variation – 0.7127

As per the FENE theory, fluid relaxes at the rate 2/3 of its strain rate representing the critical Deborah number to be around 0.66 [7], [8]. The maximum extensional viscosity around 0.66, indicating the elastic limit [8], [9] will be used as $\mu_{\max\;@De_{cr-0.66}}$ in the AT-VEM. Critical strain rate is determined by dividing the critical Deborah number by the relaxation time. The sharp increase in the elongational viscosities around the critical strain rate for the polymer solutions, used in each experiment is shown in the Figs. 2(b)–15(b). Values of $\mu_{\max\;@De_{cr-0.66}}$ for each polymer samples are shown in Table 1.

#### 2.2.3 Power law theory for strain hardening index

Power law is fitted to extensional viscosity vs strain values around the critical Deborah number for determining the *n*_*2*_ values. The power law fits for all the experiments are shown (Figs. 2(c)–15(c)). The *n*_*2*_ values determined using the power law fit are reported in Table 1.
